# Successful Management of IT Projects in Healthcare Institutions after COVID-19: Role of Digital Orientation and Innovation Adaption

**DOI:** 10.3390/healthcare10102005

**Published:** 2022-10-12

**Authors:** Elena Roxana Tucmeanu, Alin Iulian Tucmeanu, Madalina Gabriela Iliescu, Justyna Żywiołek, Zahid Yousaf

**Affiliations:** 1 Department of Management, Faculty of Medicine, Ovidius University of Constanta, 900527 Constanta, Romania; 2 Department of Management, Athenaeum University of Bucharest, 020223 Bucharest, Romania; 3 Techirghiol Sanatorium Rehabilitation Department, Faculty of Medicine, Ovidius University of Constanta, 900527 Constanta, Romania; 4 Department of Production Engineering and Safety, Faculty of Management, Czestochowa University of Technology, 42-200 Czestochowa, Poland; 5 Higher Education Department, Government College of Management Sciences, Mansehra 23100, Pakistan

**Keywords:** successful management, healthcare institutions, COVID-19, digital orientation, innovation adaption

## Abstract

This research aims to examine specific issues that how healthcare institutions successfully manage IT projects after the deadly disease of COVID-19. The world’s healthcare institute changed its traditional way of treatment to IT-based equipment after COVID-19. Hence, this study investigated the how digital orientation helps healthcare institutes for successful management of IT. Our study identifies the critical role of digital orientation and innovation adaption in the successful management of IT. The mediating role of innovation adaption in the association between digital orientation and successful management of IT was also investigated. In total, 456 questionnaires were used for the collection of data from eight different healthcare centers. We selected participants through random sampling. Findings on the healthcare institution showed that successful management of IT is predicted through digital orientation. This study’s results proved that digital orientation impacts innovation adaption, and similarly, innovation adaption influences the successful management of IT. The outcomes show the mediating role of innovation adaption in the linkage between digital orientation and successful management of IT. Current research contributes to the existing literature through combined impacts of the digital orientation, innovation adaption, and successful management of IT through means of demonstrating how, when, and why digital orientation supports the successful management of IT. Moreover, innovation adaption performs a significant role in the extant digitalize world; thus, we chose innovation adaption as a mediator in this study.

## 1. Introduction

The healthcare institutions are one of most flourishing sectors of any economy, which perform a vital role in uplifting the country’s economic and digital growth [1]. After COVID-19, the healthcare institutions are more inclined towards digitalization. Healthcare institutions involved in the successful management of IT projects need to focus not only digital orientation abilities but also need to emphasize the adaption of the innovation. The innovation adaption concept has garnered great scholarly attention since it was recognized as a key determinant for successful IT projects’ positive outcomes in healthcare institutions [2]. Digital orientation is extremely helpful and lends direction to healthcare institutions in designing and planning appropriate strategies to achieve their objective in an effective way [3]. Digital technologies and digital orientation are pertinent for the successful management of IT programs in healthcare institutions and also to improve performance [4]. Digital orientation is the deliberate ability to focus on market changes that healthcare institutions adapt, such as robotics, digitalized machinery, measures, and tools to achieve IT benefits [2,3]. Digital orientation is supported in vastly aligned research and development activities/practices that facilitate exploring innovative ideas, information, approaches, and knowledge, which healthcare institutions have used for modern technology adaption [5]. After the COVID-19 pandemic outbreak, all sectors, particularly healthcare institutions, radically changed their traditional methods due to unexpected deaths of doctors, nurses, and technicians, occurring as a result of contact with viral COVID-19 virus patients during checkup, blood test, giving/receiving test reports, etc. Accordingly, after the COVID-19 pandemic, healthcare institutions have explored tools and software applications that robotically take blood from patients and prepare test reports automatically through advanced applications without human involvement [6]. Management of healthcare institutions is mainly dependent on digital orientation ability with a view to deal with shifting requirements and to find out techniques that fulfill the prerequisites of excellence [7]. Innovation adaption refers to the implementation of creative ideas, new technologies, and knowledge with proper strategic planning to enhance productivity and increase output with similar input [5]. Accordingly, healthcare institutions that have high digital orientation ability are greatly reliant on its competence to facilitate inventiveness and become involved in practices that lead toward innovation adaption [8]. A healthcare institution’s ability to adopt changes and its knowledge about the latest technologies direct it toward innovativeness [9]. Current research involves examination to detect that how healthcare institutions can successfully manage IT projects through means of innovation adaption plus digital orientation. After the deadly disease of COVID-19, accomplishment of an ideal performance level seems to be a very tricky task; furthermore, maintaining innovativeness is also a costly and difficult job [10]. At the present time, healthcare institutions that want to deal with market orientations and reforms should devise strategies for the long term [11]. In developing nations’ healthcare institutions, it is necessary to not only deliberate but also have a proactive response in turn to fulfill the vibrant needs of market [12]. For addressing all these barriers, our study focused on the digital orientation capacity of healthcare institutions in obtaining information relating to the market and latest technology. Although they consecutively ensure competitiveness for the healthcare institutions, both digital orientation and innovation adaption also play a strategic role. The prior researches carried out so far do not provide any considerable explanation about the significant role of the digital orientation along with market orientation with respect to attaining successful management of IT through the support of innovation adaption, which constitutes a notable research gap. Previous studies enlighten several outcomes of digital orientation in other sectors, such as antecedents of supporting SMEs performance [13], roles in external environment [14], etc. After the COVID-19 epidemic, an unprecedented surge has been raised in the latest digital technologies’ usage because of social distancing customs and lockdowns all over the world. People all around the globe used digitalized applications to perform their duties and do work from home due to spread of deadly COVID-19. Digital transformation, for instance, IOT, artificial intelligence, and machine learning, presents secure and useful information-control system opportunities that healthcare institutions used for shifting toward a digital domain as a part of their transformational efforts. Digital technologies provide applications that help in designing secure authenticate certificates, medical prescriptions, records. and health test reports, etc.; such mechanisms will be important for offering such benefits. Thus, key findings present variations among the performance of healthcare institutions that used the latest digital technologies to obtain the benefits of IT competencies and those that did not restructure traditional patterns.

This research will give a platform to healthcare institutions to review the efficiency of their digital orientation ability with an outlook to attain successful management of IT. This study’s theoretical model presents four hypotheses: firstly, H1 proposes that in a healthcare institution, the digital orientation is directly associated with successful management of IT. Secondly, it is an open secret that the current global economy with an unprecedentedly growth rate is being reshaped, and thus, we explore how digital orientation plays a supportive role in the accomplishment of innovation adaption and, similarly, innovation adaption’s vital role in the successful management of IT. Thirdly, we investigate innovation adaption’s mediating role in the linkage between digital orientation and successful management of IT. In contemporary era, in every walk of the life, competitive benefits can be acquired through the application of the latest software and computer-based programs, particularly in healthcare institutions, where the latest applications and computer programs are utilized for surgical activities, lab diagnostic process, open-heart surgical actions, and pharmaceutical processes, etc. This technology usage has changed all traditional patterns. It is thus very important that the healthcare institutions are given whole patronage from the government of developing nations so that developments in various economy sectors are attained. This alteration in digital orientation for supporting healthcare institutions is more profound in developing countries. Therefore, our study suggests a more interesting model that gives significant implications for management and also recommends further funding research that highlights the corresponding changes and rapid shifts. 

## 2. Literature Review

### 2.1. Digital Orientation Successful Management of IT

Digital orientation is the premeditated strategic position in which institutions allocate their funds and resources for successful management of the IT projects to enjoy the fruitfulness of the latest technological innovation and to become trend setters through introducing novel procedures and tools [15]. Healthcare institutions that have a high digital orientation ability re able to adopt technological advancements in the industry, and they can also affect the successful management and improvement of IT projects [16]. Previous studies on healthcare institutions found that digital orientation has a positive impact on the strategic performance of a healthcare institution [17]. Despite the fact that digital orientation has also performed a significant role in the successful management of IT programs for a long time, the earlier researchers gave less value to the linkage that exists between digital orientation and the successful management of IT. Those healthcare institutions that have a higher degree of digital orientation ability reap benefits from the rapid changes taking place within IT because they have a greater competence to fulfill their patients’ requirements through new measures, machines, and techniques [18]. Digital orientation is a strategic, guiding principle for healthcare institutions along with integrating IT advancements to obtain patients’ satisfaction and maintain their profit and successful implementation of IT opportunities [19]. Healthcare institutions with the latest technological applications of software programs, graphics, and animation upgrade their planning model and have the capability to foresee and successfully manage IT advancements to enhance the routine and performance of staff, nurses, and doctors after the COVID-19 pandemic [20]. Digital orientation supports, in the long term, the success of those institutions that adopt the latest IT software and programs in competing in the market, and pursuing digital technology enables opportunities for the provision of novel machines, tools, and procedures [21].

**H1.**  *Digital orientation is positively associated with successful management of IT.*

### 2.2. Digital Orientation and Innovation Adaption

Digital orientation is a platform for healthcare institutions to learn digital processes, i.e., a preliminary step for adapting innovations. When healthcare institutions are inclined toward digital orientation for designing novel approaches and using the latest techniques, the extent of sustainable innovation adaption is raised in a healthcare institution [22]. The post-COVID-19 medical treatment is an example, as doctors and nurses have changed the way of testing and generating reports for COVID patients. Less paper work is proffered, and online test reports are delivered to patients by healthcare institutions. All these mechanisms become convenient because of the digital orientations and innovation adaption skills of healthcare institutes. Digital orientation acts as a guiding principle for healthcare institutions that creates investments in research and development activities and garners benefits from the latest refined technologies through adaption of abrupt technological advancements and novel innovation techniques [23]. Digital orientation reflects the behavioral propensity toward digital activities, such as the high-efficiency trend of digital technology innovation and the novelty and dynamic nature resulting from the utilization and incorporation of the rising digital technologies. These transformations force healthcare institutions toward the adaption of innovation [24]. Digital orientation integrates extant digitalization themes with IT research alignment for technological advancements, which lead toward introduction of different tools and measures with the help of the latest technology applications. This digitalization would lead toward technological innovation [25]. For example, in healthcare institutions, technicians, physicians, laboratory assistants, and nurses are extensively burdened with the responsibility to perform different tasks. Different doctors have to be pre-scheduled for patients’ appointments and medical procedures such as tests, X-rays, etc. If all these processes are manually performed, it often takes time for task execution and for the documentation of reports to be written. Accordingly, various errors occur due to lack of resources, pressure, shortage of time and technical equipment, etc., and the patient might have to wait; the opposite can occur if a healthcare institution has digital orientation ability that provides guidelines for focusing on the radical changes occurring in market. Digital orientation provide awareness about the latest tools, equipment, software, and applications to be adapted for observation, action, history, report, and reasoning performed by service providers during the check-ups of patients [21]. Thereby, it is important for healthcare institutions to use the latest IT technologies. Earlier studies also discussed the positive effect of digitalization on innovation models [26]. Currently, researchers broadly think that an organization’s success is mainly dependent on the digital orientation of acquisitive digital technologies, which reflects on the healthcare institution’s ability to adopt, select, update, and identify the latest advancements that meet staff requirements and give satisfaction to customers [27]. This study found that the high capacity of digital orientation facilitates agile reactions to the sustainable challenges and the adaption of the latest digital intelligence software and applications [28]. Digital orientation is the motive of healthcare institutions and the attitude of the management regarding the implementation and management of novel technological advancements with a perspective to make connections to the market through the help of the latest technological and innovative procedure adaptions for provision of good services [29]. Digital orientation aids healthcare institutions in the proactive research and development processes and supports in adapting to the latest technological applications for the augmentation of innovation processes, which are mostly supportive in innovation adaption [30].

**H2.**  *Digital orientation is associated with innovation adaption.*

### 2.3. Innovation Adaptionand Successful Management of IT

Innovation adaption provides support in the formation of novel digital products, transformation of the structure models and operational services, etc. [22]. Thus, successful management of IT programs occurs through a mixture of communication, computing, connectivity, and information technologies. Innovation adaption is at the core of developing value [31]. Innovation adaption involves in the implementation of the latest tools, knowledge, techniques, and skills that support the successful management and fulfillment of the requirements of an IT project [32]. Healthcare institutions that are looking for the latest technical systems give the highest significance to the latest advancement adaptions in their scheme to reach their technical objectives for the provision of innovative agility services through novel measures and tools [33]. Such healthcare institutions make use of these emerging developments and detailed plans for successful implementation of IT projects and the acquisition of measurable results [34]. Innovation adaption supports the management of IT projects through implementing the latest innovative technologies and techniques [35]. Successful management of IT programs require highly experienced professionals who are dedicated to the project and use their best technical knowledge and skills [36]. Innovation adaption is linked with systematic planning, monitoring, implementation, and revision of the latest IT channels between all concerned professionals in the project through adaption of the innovative means [37]. Healthcare institutions that highly emphasize the acquisition of new innovative technology and purposefully maintain relationships with relatable stakeholders, such as research and development and competitor healthcare institutions, in turn adopt the latest innovative technology trends for successful management of IT programs [38]. Therefore, from the above discussion, we obtain the hypothesis that

**H3.**  *Innovation adaption is linked with successful management of IT.*

### 2.4. Innovation Adaptionas Mediator

One of the imperative goals of this research is to explore innovation adaption’s mediating role pertaining to digital orientation vis-à-vis successful management of IT [39]. Technology-driven healthcare institutions give the latest updated news and information concerning advancements, besides what is profoundly covered in research and development processes, to strengthen innovation adaption practices [40]. Healthcare institutions having high levels of digital orientation are involved in dealing with technology businesses that gather innovative information through investing a heavy budget into research and development process [41]. They make investments in the latest technology and provide solid fundamentals for the adaption of innovation [42]. If a healthcare institution’s adaption of innovation corresponds in a correct way to its implementation at the appropriate time, it leads to high profit and successful management of IT programs [43]. The first mover’s innovation adopters may effortlessly develop long-term connections with their patients or customers that will positively affect their healthcare institution’s competitiveness [44]. Healthcare institutions in the developing nations have to focus on digital orientation so that innovation adaption is protected, which is a precondition for the successful management of IT projects [45]. It is a renowned secret that successful management of IT projects is greatly dependent on innovative thinking and experts’ technical skills [46]. Digital orientation provides awareness about the latest technological trends, which eventually determine innovation adaption [47]. Therefore, our study explores the impact of the digital orientation on innovation adaption through the integration of innovation adaption as a mediator. Thus, this study aims to expose innovation adaption’s mediating role on the linkage between digital orientation and successful management of IT. Healthcare institutions that have a high digital orientation ability obtain innovative information, which might result in high profitability and can be used afterward for successful management of IT projects [48].

**H4.**  *Innovation adaption mediates the association between digital orientation and successful management of IT.*

**Figure 1:**
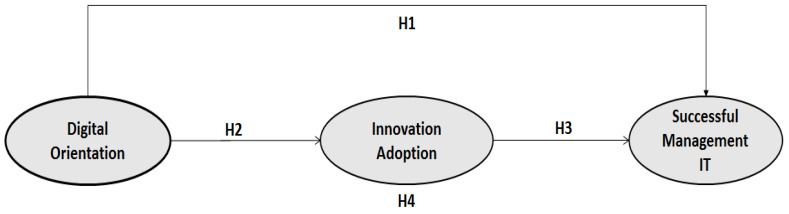
Digital Orientation on Successful Management of IT.

Figure 1 shows the theoretical model.

## 3. Methodology

The current research is quantitative in nature, in which a survey was carried out through questionnaire. For this research, the investigation element is healthcare institutions. After COVID-19, the healthcare institutions of Pakistan rapidly changed their traditional patterns, where modification and novel developments took place unprecedentedly. We considered IT projects that involved strategic planning concerning investment in the restructuring of labs machinery and equipment, doctors’ treatment procedure, and comprehensive plans regarding software and app adaption/installations. IT-based projects are multi-factor strategies that support training sessions given to technicians, lab assistants, receptionists, and nurses relating to the use of the digital technologies and achievement of IT goals in healthcare institutions. We selected participants through random sampling from hospitals from eight different healthcare centers. For data collection, healthcare professionals, i.e., doctors, surgeons, technicians, and nurses, that fulfilled the appropriate profile requirements, such as having comprehensive information about organization and also participating in several kinds of IT project implementations, were selected as respondents to reply the questionnaire. Questionnaires were distributed through the help of three research associates through email to the respondents. A total of 456 questionnaires was distributed among respondents, in which only 350 questionnaires were returned back having completed the study requirements and were used for further analysis. The total data collection process took one month and yielded a return rate of 83.3%. 

The questionnaire was divided into two sections: Section 1 contains demographic variables such as age, nationality, job experience, education, and position. In Section 2, all study items were enlisted. 

### 3.1. Measurement

For the measurement of study constructs, questionnaire items were strictly developed and checked by academic experts to ensure their reliability and accuracy. The study items were adapted from prior studies and tested on a 5-point Likert scale, in which 0 = strongly agree, and 5 = strongly disagree. 

### 3.2. Digital Orientation

For the measurement of digital orientation, a 7-items Likert scale was used, which was adapted from [49]; however, we also obtained guidelines from [50]. 

### 3.3. Innovation Adaption

Innovation adaption was measured through a 7-item scale, which was used in prior studies and adapted from [51,52]. 

### 3.4. Successful Management of IT

The successful management of IT was measured through a 5-item scale, which was adapted from [53], but we also studied and obtained good guiding principles about these items’ design from [54].

## 4. Analysis

Table 1 shows the results of factor loading average variance extract (AVE), Cronbach’s alpha (α), convergent validity, and composite reliability (CR). All the results were reliable and valid. Discriminant validity was accepted with the test of [55], and factor loading was greater than 0.70, while average variance extract was higher than 0.50. Composite reliability was above 0.60, and Cronbach’s alpha (α) was greater than 0.70.

Table 2 shows the CFA for model fitness, and results proved that our final model, the fourth model, is appropriate to the data (χ^2^ = 1065.52, df = 465; χ^2^/df = 2.291; RMSEA = 0.05; CFI = 0.96; GFI = 0.95).

Table 3 indicates the results of mean, standard deviation, and correlation. Digital orientation (DO), innovation adaption (IA), and successful management of IT were evaluated. DO is significant and positively associated with SMIT (r = 0.28 **, *p*-value = significant). Innovation adaption is positively associated with SMIT (r = 0.38 **, *p*-value = significant). DO and innovation adaption have a significant effect (r = 0.32 **, *p*-value = significant). The VIF scores for healthcare institutions also indicate that multi-collinearity is not an issue in this research, as its values were less than 10.0.

Table 4 show the results of our hypotheses: H1 proposed that digital orientation positively affects SMIT(β value = 0.26 **;H1 is proven *p* = significant). Digital orientationis positively affected by innovation adaption (β value = 0.34 **, *p* = significant) H2 is therefore proven. Innovation adaption is significantly impacted by SMIT (β value = 0.32 **, *p* = significant), and thus, H3 is accepted.

Table 5 shows the mediating effect of innovation adaption between digital orientation effort and successful management of IT. The test analysis was used with a 5000 bootstrap method at 95% confidence level [56,57]. Table 5 also indicates the results of the indirect impact of DO on SMIT through innovation adaption (DO→IA→SMIT). Table 5 presents all the paths’ descriptions, i.e., total, direct, and indirect paths effect): ‘a, b, c, c’, and ab’). Path ‘a’ proved DO’s effect on innovation adaption (B = 0.5725, t = 7.5781, *p* = sig). Path ‘b’ proved innovation adaption’s direct effect on SMIT (B = 0.3674, t = 7.1245, *p* = sig). Path ‘c’ proved the total effect of DO on SMIT (B = 0.2674, t = 3.7415, *p* = sig). Path ‘c’ explains that when innovation adaption was measured, the direct effect of DO on SMIT was compact and non-significant, proving the mediating effect (B = 0.1825, t = 1.3567, *p* = 0.1312). Path ‘ab’ shows the results of an indirect effect in the last portion of Table 5. The results of the indirect effect proved that innovation adaption performs as a mediator (B = 0.16, lower = 0.105 to upper = 260. Thus, H4 was proven, and it is proven that the link between DO and SMIT is mediated through innovation adaption.

## 5. Discussion

This research aims to investigate the determinants that influence successful management of IT projects, such as digital orientation and innovation adaption. Summing up the set of the comprehensive empirical model, our study primarily presents four hypotheses and thus suggests significant practical and theoretical implications. In this study, H1 demonstrates the direct effect of digital orientation on the successful management of IT. Our study offers insight about how a representative group of nurses, doctors, and technicians perceive new technology and successfully manage IT programs after the COVID-19 pandemic. This study’s findings support the previous scholars’ findings that digital orientation is the premeditated, strategic position in which institutions allocate their funds and resources for successful management of IT projects to enjoy the fruitfulness of the latest technological innovations and become trend setters through introducing novel procedures and tools [15]. Healthcare institutions with high digital orientation ability are able to adopt technological advancements in the industry, and they can also affect the successful management and improvement of IT projects [16]. Previous studies on healthcare institutions found that digital orientation has a positive impact on the strategic performance of a healthcare institution [17]. Despite the fact that digital orientation has performed a significant role in the successful management of IT programs for a long time, the earlier researchers gave less value to the linkage that exists between digital orientation and successful management of IT. Those healthcare institutions that have a higher degree of the digital orientation ability obtain benefits from the rapid changes taking place within IT because they have greater competence to fulfill their patients’ requirements through new measures, machines, and techniques [18]. Digital orientation is a strategic, guiding principle for healthcare institutions along with integrating IT advancements to obtain patients’ satisfaction, maintain their profit, and successfully implement IT opportunities [19]. Healthcare institutions with the latest technological applications of software programs, graphics, and animation upgrade their planning model and have the capability to foresee and successfully manage IT advancements to enhance the routine and performance of staff, nurses, and doctors after the COVID-19 pandemic [20]. The outcomes for healthcare institutions are that digital orientation is essential for healthcare institutions, and digital orientation positively and considerably affects successful management of IT. In the current research, H2 proposed that digital orientation directly influences innovation adaption. Technology-driven healthcare institutions alter traditional patterns and adopt new innovative technological tools in wide ranges for their healthcare settings. The results are consistent and support previous studies’ findings that when healthcare institutions are inclined toward digital orientation for designing novel approaches and using the latest techniques, the extent of sustainable innovation adaption is raised in healthcare institutions [23]. Digital orientation acts as a guiding principle for healthcare institutions that create investments in research and development activities and obtain benefits from the latest refined technologies through adaption of abrupt technological advancements and novel innovation techniques [24]. Digital orientation integrates extant digitalization themes with IT research alignment for technological advancements, which lead toward the introduction of different tools and measures with the help of the latest technology applications. This digitalization would lead toward technological innovation [25]. Earlier studies also discussed the positive effect of digitalization on the innovation model [26]. Organizations’ success is mainly dependent on the digital orientation of acquisitive digital technologies. It enhances healthcare institutions’ ability to adopt, select, update, and identify the latest advancements that meet staff requirements and give satisfaction to customers [27]. This study found that a high capacity of digital orientation facilitates agile reactions to sustainable challenges and the adaption of the latest digital intelligence software and applications [28]. Digital orientation is the motive of healthcare institutions and the attitude of the management regarding the implementation and management of novel technological advancements [29]. Thus, we hypothesized that high digital orientation provides awareness about the adaption of new innovative trends and tools. Furthermore, in this research, H3 explores how innovation adaption positively affects successful management of IT.

In our study, we investigated the perception of nurses about innovation adaption and asked them what they emphasized regarding the use of the latest technological applications. Innovation adaption provides support in the formation of novel digital products, transformation of the structure models and operational services, etc., and thus successful management of IT programs. Innovation adaption is at the core of developing value [31]. Innovation adaption involves the implementation of the latest tools, knowledge, techniques, and skills that support the successful management and fulfillment of the requirements of an IT project [32]. Healthcare institutions that are using technology-driven systems give the highest significance to the adaption of the latest advancements [33]. Such healthcare institutions make use of these emerging developments and detailed plans for successful implementation of IT projects and the acquisition of measurable results [34]. Innovation adaption supports the management of IT projects through implementing the latest innovative technologies and techniques [35]. Successful management of IT programs require highly experienced professionals who are dedicated to the project and use their best technical knowledge and skills [36]. Innovation adaption is linked with systematic planning, monitoring, implementation, and revision of the latest IT channels between all concerned professionals in the project through adaption of the innovative means [37]. The findings support the conclusions regarding healthcare institutions in H3. This research paves the right way for healthcare institutions in implementing the latest methods. The findings of H4 showed that the association between digital orientation and successful management of IT is mediated through innovation adaption. Innovation adaption is a significant factor in the successful management of IT projects in healthcare institutions. However, the outcomes corroborate that technology-driven healthcare institutions generate the latest updated news and information concerning advancements, besides what is profoundly covered in research and development processes, to strengthen innovation adaption practices [40]. Healthcare institutions having a high level of digital orientation are involved in dealing with technology businesses that gather innovative information through investing a heavy budget into the research and development process [41]. They make investments in the latest technology and provide solid fundamentals for the adaption of innovation [42]. If a healthcare institution’s adaption of innovation corresponds in the correct way to its implementation at the appropriate time, it leads to a high profit and the successful management of IT programs [43]. The first mover’s innovation adopters may effortlessly develop long-term connections with their patients or customers that will positively affect their healthcare institution’s competitiveness [44]. Healthcare institutions in the developing nations have to focus on digital orientation so that innovation adaption is protected, which is a precondition for the successful management of IT projects [45]. Successful management of IT projects is greatly dependent on innovative thinking and experts’ technical skills [46]. Digital orientation provides awareness about the latest technological trends, which eventually determine innovation adaption [47]. Therefore, our study explores the impact of digital orientation on innovation adaption through the integration of innovation adaption as a mediator. Our findings proved that innovation adaption acts as a bridge between digital orientation and successful management of IT programs. Overall, our study gives a meaningful contribution to the stream of literature knowledge and supports all direct and indirect hypotheses.

### 5.1. Theoretical Implications

From the findings of current research, a number of theoretical and practical implications can be drawn. First, we proposed that digital orientation facilitates the enhancement of traditional practices through adaption of advancements and innovative tools and measures for provision of good services. That contributes to attracting patients, motivating them, and protecting them from the harmful impacts of error in healthcare institutions. Secondly, we recommend that management should emphasize the adaption of innovative tools and procedures for the successful management of IT programs. Thirdly, this research suggests that healthcare institutions ought to have the latest technological applications, e.g., software and tools for the successful management of IT projects. When healthcare institutes use the latest technologies, it will assist them in the successful management of IT programs, such as lab development, technicians’ training, and medical treatment procedures. Lastly, today, computer technologies’ adaption, such as artificial intelligence, machine learning, and cloud computing, performs a critical role in the achievement of innovation that particularly leads toward successful management of IT programs through a supplemented means of digital orientation. The cooperation of the advanced technological applications along with innovative practices will facilitate the successful management of IT.

### 5.2. Practical Implications

This study offers some significant practical implications to administration and management. Firstly, we propose that innovation adaption is the vital factor in acquiring, developing, and designing techniques and measures that facilitate the successful management of IT through the antecedents of digital orientation. Secondly, we suggest that digital orientation is a guiding principle for the adaptation of innovative technological applications, and similarly, innovation adaption supports the successful management of IT programs. Lastly, healthcare institutions should focus on the improvement of digital orientation abilities as a strategic resource to augment the potential for implementation of the latest applications that lead toward successful management of IT projects.

### 5.3. Limitations and Directions for Future Research

This study has several boundaries that may be considered as future direction for other studies. Firstly, we considered successful management of IT as a dependent construct and investigated the determinants that affect their performance. In the future, other studies should use other constructs and investigate their determinants. Secondly, we examined this study setting based on the quantitative data; in future research, a qualitative approach may be used. Thirdly, this research was carried out on healthcare institutions, and in the future, a study could be conducted on some other sector with this empirical model. Finally, the current study explored the mediating role of innovation adaption, while other studies may be used to explore other constructs.

## Figures and Tables

**Table 1:** Results of Cronbach’s alpha, CR and AVE.

Variable Detail	Items	Fac-L	T-Value	Alpha	CR	AVE
**Digital Orientation**				0.82	0.94	0.76
DO-1	In our healthcare institution: We adapt the latest digital technology for the achievement of corporate and communication efficiency.	0.82	15.54			
DO-2	We implement digital machines to overcome research and development costs.	0.85	15.21			
DO-3	We bring into play the latest digital technologies for the management of information, such as patient, staff, and building, etc.	0.81	14.54			
DO-4	Our goal is to deliver new test and treatment services through digital technology.	0.78	15.74			
DO-5	Our goal is to adopt novel patterns of working.	0.88	15.63			
DO-6	We used advanced digital tools to make linkages with our stakeholders and patients.					
DO-7	We used emerging technology for developing a reasonable and beneficial structure model.	0.82	14.63			
**Innovation Adaption**				0.88	0.92	0.72
IA-1	In our healthcare institution: We adopt clear guidelines and procedures for achieving innovation.	0.86	15.47			
IA-2	We observe that innovation techniques that we adopt are beneficial for health professionals.	0.82	14.52			
IA-3	We find relevance degree of innovation tool for patient.	0.76	15.74			
IA-4	We identify staff capacity in implementation of innovation in the hospital.	0.74	15.56			
IA-5	We investigate how many experts or specialist exists that work on the latest innovative machines.	0.84	14.21			
IA-6	We identify benefits and patient willingness to collaborate with innovation.	0.82	15.48			
IA-7	We analyze the extent to which the financial burden of innovation is imposed on patients.	0.76	14.75			
**Successful Management IT**				0.86	0.98	0.74
SMIT-1	Project management success is evaluated through deviations occurring after plan implementation.	0.84	15.74			
SMIT-2	For project management, a successful solution is implemented in accordance with the budget.	0.76	14.53			
SMIT-3	For successful management of IT projects, a system should have high reliability.	0.82	15.48			
SMIT-4	For project success, solutions should meet requirements and contribute to efficiency.	0.78	14.52			
SMIT-5	IT systems work and solve the problem and also are profitable for our healthcare institution.	0.74	14.64			

**Table 2:** Healthcare Institutionary Factor Analysis (CFA).

Model Description	χ^2^	Df	χ^2^/df	RMESA	GFI	CFI
Model-4-Factor(Hypothesized)	1065.52	465	2.291	0.05	0.95	0.96
Model-3-Factor	1145.23	390	2.936	0.13	0.85	0.86
Model-2-Factor	1295.47	375	3.455	0.18	0.74	0.75
Model-1-Factor	1490.36	355	4.198	0.22	0.65	0.66

**Table 3:** Correlations.

Variable		Mean	SD	Alpha	1	2	3	4	5	6	7
1	Business-Age	3.00	1.06	0.85	1.00						
2	Business-Size	1.28	0.41	0.84	1.62 **	1.00					
3	Respondent-Education	1.54	0.48	0.81	0.014	0.036	1.00				
4	Respondent-Experience	1.25	0.42	0.85	0.035	0.047	−0.142	1.00			
5	Digital Orientation	3.17	0.34	0.86	0.104 **	0.016	0.024	−0.11	1.00		
6	Innovation Adaption	3.46	0.44	0.82	−0.029	0.082 *	0.056 **	−0.12	0.325 **	1.00	
7	Successful Management of IT	1.25	0.35	0.83	0.021	0.001	−0.03	−0.03	0.283 **	0.385 **	1.00

Note: ** = significant at 0.001 and * = significant at 0.05.

**Table 4:** Hypothesis Testing (H1,H2,and H3).

Model	Hypothesis Description	B	F	T	Sig	Remarks
Model # 01	Digital Orientation to Successful Management of IT	0.26	16.058	0.1245	0.000	Accepted
Model # 02	Digital Orientation to Innovation Adaption	0.34	18.054	0.1045	0.000	Accepted
Model # 03	Innovation Adaption to Successful Management of IT	0.32	22.756	0.1454	0.000	Accepted

**Table 5:** (Mediating Effect of Innovation Adaption between Digital Orientation and Successful Management of IT.

Paths Description			Beta	T-Value	SE	Sig
**Digital Orientation to Innovation Adaption (Path a)**			0.5725	7.5781	0.0524	0.000
Innovation Adaption to Successful Managementof IT (Path b)			0.3674	7.1245	0.0412	0.000
Digital Orientation to Successful Management of IT (Path c)			0.2674	3.7415	0.0554	0.000
Digital Orientation to Successful Management of IT (Path c’)			0.1825	1.3567	0.0557	0.1312
*DV Model: R^2^* = *0.1391*, *F = 34.7321*, *p = 0.000*						
**Detail**	**Data**	**Boot**	**SE**	**Lower**	**Upper**	**Sig**
DO→IA→SMIT	0.16	0.17	0.32	0.105	0.260	0.0000

## Data Availability

Not applicable.
